# Genetic hitchhiking in a subdivided population of *Mytilus edulis*

**DOI:** 10.1186/1471-2148-8-164

**Published:** 2008-05-30

**Authors:** Matthieu F Faure, Patrice David, François Bonhomme, Nicolas Bierne

**Affiliations:** 1 Université Montpellier II, Place Eugène Bataillon, 34095 Montpellier, France; 2 CNRS – Institut des Sciences de l'Evolution UMR5554 Montpellier, France; 3 CEFE – CNRS, 34293 Montpellier Cedex 5, France; 4 Département de Biologie Intégrative, Institut des Sciences de l'Evolution – UMR5554, Station Méditerranéenne de l'Environnement Littoral, 1 Quai de la Daurade, 34200 Sète, France

## Abstract

**Background:**

Few models of genetic hitchhiking in subdivided populations have been developed and the rarity of empirical examples is even more striking. We here provide evidences of genetic hitchhiking in a subdivided population of the marine mussel *Mytilus edulis*. In the Bay of Biscay (France), a patch of *M. edulis *populations happens to be separated from its North Sea conspecifics by a wide region occupied only by the sister species *M. galloprovincialis*. Although genetic differentiation between the two *M. edulis *regions is largely non-significant at ten marker loci (average F_ST_~0.007), a strong genetic differentiation is observed at a single locus (F_ST _= 0.25). We validated the outlier status of this locus, and analysed DNA sequence polymorphism in order to identify the nature of the selection responsible for the unusual differentiation.

**Results:**

We first showed that introgression of *M. galloprovincialis *alleles was very weak in both populations and did not significantly affect their differentiation. Secondly, we observed the genetic signature of a selective sweep within both *M. edulis *populations in the form of a star-shaped clade of alleles. This clade was nearly fixed in the North Sea and was segregating at a moderate frequency in the Bay of Biscay, explaining their genetic differentiation. Incomplete fixation reveals that selection was not direct on the locus but that the studied sequence recombined with a positively selected allele at a linked locus while it was on its way to fixation. Finally, using a deterministic model we showed that the wave of advance of a favourable allele at a linked locus, when crossing a strong enough barrier to gene flow, generates a step in neutral allele frequencies comparable to the step observed between the two *M. edulis *populations at the outlier locus. In our case, the position of the barrier is now materialised by a large patch of heterospecific *M. galloprovincialis *populations.

**Conclusion:**

High F_ST _outlier loci are usually interpreted as being the consequence of ongoing divergent local adaptation. Combining models and data we show that among-population differentiation can also dramatically increase following a selective sweep in a structured population. Our study illustrates how a striking geographical pattern of neutral diversity can emerge from past indirect hitchhiking selection in a structured population.

**Note:**

Nucleotide sequences reported in this paper are available in the GenBank™ database under the accession numbers EU684165 – EU684228.

## Background

The detection of adaptive evolution at the molecular level has essentially relied on indirect inferences [1]. This is simply because genomes are very large and adaptive evolution probably occurs only at a few segregating mutations at a given time [2]. However, adaptive evolution leaves a footprint on the pattern of neutral diversity [3], which widens both the genomic extent and the time scale on which adaptation can be detected. The theory is very well developed in the case of a single panmictic population. The hitchhiking model of Maynard Smith and Haigh [3] predicts that the fixation of an advantageous mutation decreases the diversity at linked neutral loci. The effect of a so-called selective sweep on the allele frequency spectrum [4], and more generally on gene genealogies [5,6] is also well established. Along with the development of a battery of statistical tests [7], empirical examples have accumulated [8-12]. Although the indirect path through which selection shapes genetic diversity bears many resemblances to demographic effects [13,14], selection only acts on the chromosomal neighbourhood of the site targeted by selection while demography affects the whole genome [15,16]. The recent development of genome scans now allows appreciating how genomes seem crippled by numerous signatures of adaptive evolution [17,18].

Most models describe the spread of an advantageous mutation in a single isolated population and examples of genetic hitchhiking remain bordered on such an idealised model. However, natural populations are most of the time structured into geographically or genetically partially isolated populations [19]. The hitchhiking effect in structured populations has been less intensively investigated. Few models have been developed [20-23] and they sometimes give contradictory results. Although a reduction of genetic diversity at the metapopulation level is a robust expectation, the effect of a selective sweep on the distribution of the genetic diversity within and between populations is less clear. Although statistical tests have been developed in order to identify loci showing more or less population differentiation than predicted under neutrality [24-27], the exact form of the selection responsible for extreme differentiations is hardly ever addressed. Indeed, loci with a higher than expected F_ST _are simply assumed to be under divergent selection ('local adaptation') while loci with a lower than expected F_ST _are assumed to be under balancing selection. In addition, the question of whether selection acted directly on the polymorphism observed or indirectly through genetic hitchhiking is often eluded [28]. Regrettably, it has seldom been noticed by experimentalists that the hitchhiking effect of an unconditionally favourable mutation that spread from its deme of origin to other demes by migration ('hitchhiking in space' [29]) can sometimes enhance population differentiation as measured by F_ST _[21]. Few studies have attempted to investigate more explicitly how selection has operated on an outlier locus by examining DNA sequence variation [30]. Albeit some genome scans were recently conducted with DNA sequence polymorphisms in humans and flies [17,31], these studies emphasised the occurrence of hitchhiking within derived populations thought to have recently adapted to a new environment (i.e. the 'local adaptation' hypothesis).

**Figure 1:**
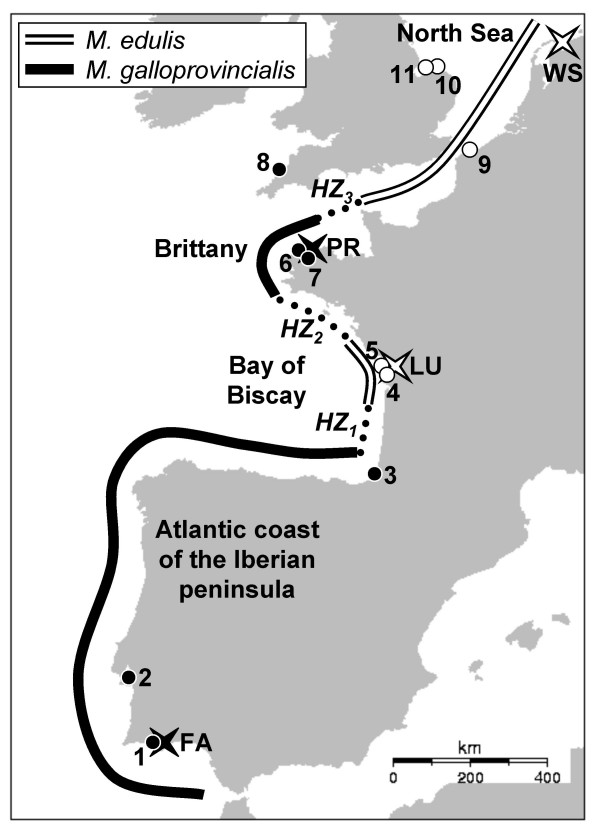
**Localities of *Mytilus *spp. samples along the European coast**. The four samples chosen to be representative of each panmictic species patch and used for DNA sequence analysis are indicated by white and black stars for *M. edulis *and *M. galloprovincialis *respectively. Additional previously analysed samples [33] used for the correspondence analysis are indicated by white and black dots for *M. edulis *and *M. galloprovincialis *respectively. Sample names and sample sizes (in brackets) are the following: 1, Faro (67); 2, Setubal (28); 3, Biarritz (50); 4, Brouage (30); 5, Boyard (50); 6, Moguériec (24); 7, Morlaix (24); 8, Polzeath (49); 9, Grand-Fort-Philippe (50); 10, Tichwell (27); 11, Cley (32). The three hybrid zones [33] are indicated by dashed lines: *HZ*_1 _between *M. galloprovincialis *of the Atlantic coast of Iberian Peninsula and *M. edulis *of the Bay of Biscay, *HZ*_2 _between *M. edulis *of the Bay of Biscay and *M. galloprovincialis *of Brittany and *HZ*_3 _between *M. galloprovincialis *of Brittany and *M. edulis *of the North Sea. Patches of *M. galloprovincialis *are indicated with continuous black lines and patches of *M. edulis *are indicated by black and white continuous lines.

The objective of the present study was to identify the nature of the selection (positive *vs*. disruptive, direct *vs*. indirect, past *vs*. ongoing) acting on a locus with a higher than expected level of population differentiation in the mussel *Mytilus edulis*. The structure of the hybrid zone between *Mytilus edulis *and *M. galloprovincialis *in Europe is an original mosaic structure in which populations of each species alternate [32,33]. Along the Atlantic coast of France, three transition zones separate four regions from North to South (Figure 1): a large Northern patch of *M. edulis *connected to the North Sea populations, a patch of *M. galloprovincialis *centred on Brittany, a patch of *M. edulis *centred on the Bay of Biscay, and a large southern population of *M. galloprovincialis *connected to Iberic Atlantic populations [33]. We noticed that populations of the two enclosed patches (Brittany, where *M. galloprovincialis *populations are surrounded by *M. edulis*; and Bay of Biscay, where the reverse is true) exhibited genetic originalities compared to their external conspecifics (southern and northern patches respectively). These allelic differences were unequally distributed among loci suggesting the possible impact of selection. Within *M. edulis*, differences between peripheral populations of the North Sea and the patch of the Bay of Biscay were restricted to a single locus named *EFbis*. *EFbis *was amplified by the Exon-Primed Intron-Crossing (EPIC) PCR technique and revealed length-polymorphisms within the third intron of the Elongation Factor 1α (*EF1α*) gene. Here, we wanted first to validate the outlier status of the *EFbis *locus. We compiled data at five allozyme loci [34], and six nuclear DNA loci [33] between peripheral populations of the North Sea and the patch of the Bay of Biscay. To better understand the unforeseen behaviour of the *EFbis *locus we investigated DNA sequence polymorphisms at a ~1 Kb portion of the *EF1α *gene that includes the third intron (*i.e*. the region analysed with length polymorphism) but also the second intron and three exons (a large portion of the second, the third, and a small portion of the fourth). We assessed whether the genetic structure observed between *M. edulis *populations could be a consequence of differential introgression of *galloprovincialis *alleles by analysing *M. galloprovincialis *samples. We looked for signatures of hitchhiking events in one or both populations in the form of star-shaped allele genealogies and using various neutrality tests. We also modelled the effect of a barrier to gene flow to better understand how population structure may interact with selective sweeps so as to produce the pattern observed.

## Results

### Is EFbis an F_ST _outlier?

**Figure 2:**
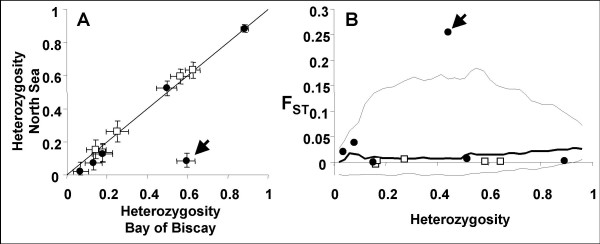
**Tests of the outlier status of locus *EFbis***. **A**: Heterozygosity in *Mytilus edulis *populations of the North Sea plotted against heterozygosity in populations of the Bay of Biscay. The 5 allozyme loci are represented by squares and the 6 DNA loci by dots. Locus *EFbis *is indicated by an arrow. **B**: F_ST _values between *M. edulis *of the North Sea and *M. edulis *of the Bay Biscay plotted against heterozygosity. Single locus F_ST _values are estimated from 5 allozyme loci (squares) and 6 nuclear DNA loci (dots). The outlier *EFbis *locus is indicated by an arrow. Average (bold line) and 95% confidence envelope (thin lines) are the results from simulations performed with the fdist2 program with the following parameters: infinite-allele model of mutation, 50 islands, two samples and the average gene flow deduced from the all eleven loci.

The heterozygosities observed in the two *M. edulis *patches were similar with the exception of the *EFbis *locus (Figure 2A). At the *EFbis *locus, heterozygosity was significantly lower in the patch of the North Sea than in the patch of the Bay of Biscay. The result of the test of Beaumont and Nichols [25] is presented in Figure 2B as a distribution of F_ST _with heterozygosity on which we superimposed single-locus F_ST_-values obtained with allozyme or length-polymorphism loci. Curves of the average F_ST _and 95% confidence intervals plotted in Figure 2 have been generated by simulations with the infinite-allele model of mutation, 50 islands (demes), two samples and the average gene flow deduced from all eleven loci. Other parameters we tried, such as setting the model to have only two demes in order to fit the idea of a two-patch model more closely, always produced narrower 95% confidence intervals (data not shown). We observed a strong and highly significant differentiation at locus *EFbis *(F_ST _= 0.255, Fisher exact test: P < 0.001) while the other ten loci exhibited very low and non-significant levels of differentiation. Locus *EFbis *appeared to be an outlier at the 95% limit when included in the test (Figure 2B). Note, that we also removed the *EFbis *locus from the test while generating the F_ST _envelope (an iterative fitting suggested by [25]). None of the 20,000 realisations produced a higher F_ST _than the one observed at the *EFbis *locus and the curve of the average F_ST _fitted single-locus F_ST_-values more closely (not shown).

### Genetic composition of samples

**Figure 3:**
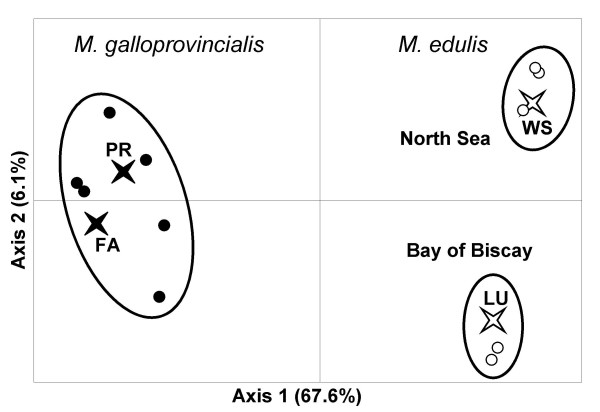
**Projection of samples centers of gravity on the first factorial plane of a correspondence analysis (CA) on the matrix of allele frequencies at three loci (*EFbis*, *Glu-5' *and *mac-1*)**. The four samples representative of each species patch used for the analysis of DNA sequences are indicated by white and black stars for *M. edulis *and *M. galloprovincialis *respectively. The eleven samples previously analyzed [33] are indicated by white and black dots for *M. edulis *and *M. galloprovincialis *respectively. Ellipses group samples for which the homogeneity of allelic frequencies could not be rejected at the *EFbis *locus.

Figure 3 shows the projection of samples on the plane defined by the first two factorial axes of the CA. Axis 1 explains 67.6% and axis 2 6.1% of the variance in allele frequencies. Axis 1 mainly reflected the allele frequency gradient between the two species, from *M. galloprovincialis *on the left to *M. edulis *on the right. The first factorial plane clearly discriminated three groups of samples (ellipses in Figure 3). Within each group, the homogeneity of genotypic frequencies could not be rejected at locus *EFbis*. Although some differences between *M. galloprovincialis *of the Iberian Peninsula and Brittany were visible on the third axis (not shown, see [33]), the homogeneity of allele frequencies was not rejected at locus *EFbis*. The three new samples (FA, LU and WS) presented a genetic composition in conformity with expectations based on their geographic location (Figure 1 and 3). Finally, as for the 11 reference samples added in the analysis [33], none of our new samples significantly departed from Hardy-Weinberg and linkage equilibrium when tested with Barton's maximum likelihood ratio test [35]. We redid the analysis of Bierne et al. [33] again with our new samples because it allows inferring that shared polymorphism observed in our samples is either a consequence of introgression or ancestral polymorphism but not a consequence of admixture (here defined as a population composed of individuals of the two species and/or first generations hybrids). In other words, the genetic composition of the samples used for DNA sequence analysis fitted their geographic position and were not polluted by admixture or recent events of hybridisation.

### Cloning

We here describe the results obtained with the mark-recapture (MR)-cloning protocol [36]. In MR-cloning a single cloning reaction is done for a population sample and each individual sequence is subsequently assigned to an individual with the two small poly-nucleotide tags that flank the primer sequences (see methods). We sequenced 185 positive clones and obtained a sequence for 64 individuals (GenBank accession numbers – Sample FA: EU684206 to EU684212, Sample LU: EU684181 to EU684205, Sample PR: EU684213 to EU684228, Sample WS: EU684168 to EU684180). Final sample sizes are reported in Table 1. The LU sample size was much bigger than the others simply because the cloning reactions worked very well and allowed us to sequence many positive clones (Table 1). When the two alleles of heterozygous individuals were obtained, only the most captured sequence was conserved for the data analysis in order to prevent sampling bias. Alleles sequenced several times allowed us to estimate the frequency of artefactual mutations inherently produced during PCR, cloning and sequencing. We found on average one artefactual mutation for ~3000 bp sequenced in our dataset. We constructed datasets exclusively composed of sequences captured several times (see methods) which should be essentially free from artefactual mutations [36]. Results obtained with the global datasets (G dataset) were compared to the results obtained with high quality subsets (HQ subsets). As *M. galloprovincialis *samples were here studied to control for introgression of *galloprovincialis *alleles in *M. edulis *populations, sample size does not need to be high for these samples and we present results obtained with HQ subsets.

**Table 1:** Sample sizes, molecular diversities and tests of neutrality

Sample name :	Dataset type	*n*	*S*	*S*_*syn*_	*S*_*ns*_	*S*_*i*_	*θ*_*W*_	θ_π_	*R*_*m*_	*D*	*H*
***M. galloprovincialis *Atlantic (FA+PR)**	HQ	9	46	4	2	40	0.017	0.013 ± 0.003	1	-1.37	-7.11
***M. edulis *Bay of Biscay (LU)**	G	24	76	8	2	66	0.017	0.014 ± 0.002	0	-0.72	-0.97
	HQ	18	58	5	1	52	0.014	0.015 ± 0.002	0	0.12	-0.78
***M. edulis *North Sea (WS)**	G	13	57	8	4	45	0.015	0.007 ± 0.003	0	-2.29***	-13.17**
	HQ	7	44	6	3	35	0.015	0.01 ± 0.005	0	-1.67**	-10.19*

G, global dataset; HQ, high quality subset exclusively composed of sequences captured several times; *n*, sample size; *S*, number of polymorphic sites; *S*_*syn*_, number of synonymous polymorphic sites; *S*_*ns*_, number of non-synonymous polymorphic sites; *S*_*i*_, number of intronic polymorphic sites; θ_*W*_, nucleotide diversity estimated from the number of polymorphic sites [68];, θ_π _nucleotide diversity estimated from the average number of pairwise differences [37] with standard deviation; *R*_*m*_, estimation of the minimum number of recombination [69]; *D*, Tajima's D [39]; *H*, Fay and Wu's H [4]. *P < 0.05, **P < 0.01, ***P < 0.001.

### Nucleotide variation

Significant genetic differentiation was observed at the *EFseq *locus with permutation test between each pair of populations except between the *M. galloprovincialis *samples of Brittany and the Atlantic coast of the Iberian Peninsula, as was already the case with intron-length polymorphism. As a consequence the two samples were pooled together and simply labelled as *M. galloprovincialis*. Basic descriptors of polymorphism are presented in Table 1. A high level of nucleotide diversity was observed. The *M. edulis *sample of the North Sea, however, exhibited a significantly lower level of nucleotide diversity (*θ*_*π*_, [37]). In addition, the polymorphism of this sample was essentially composed of rare mutations (singletons). This result logically echoed the lack of diversity obtained in the North Sea patch with length-polymorphism in the third intron (Figure 2A).

### Allele phylogeny

**Figure 4:**
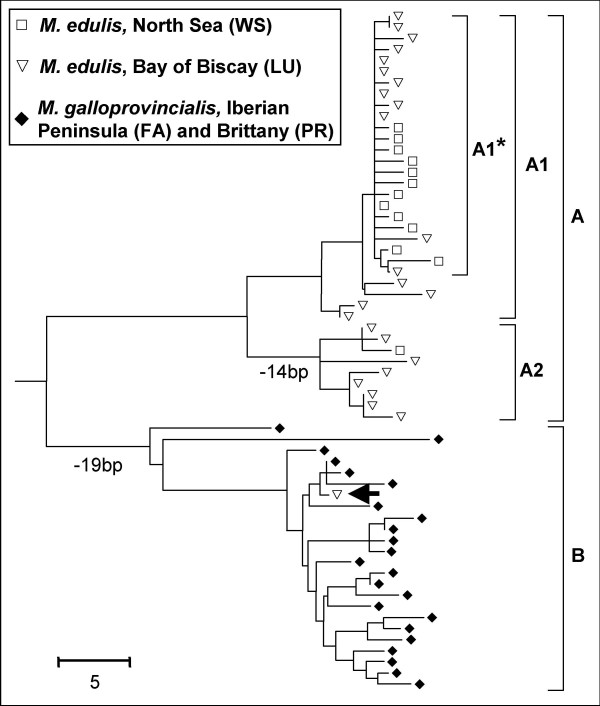
**Neighbor Joining tree on the number of nucleotide differences at the *EFseq *locus**. Sequences sampled in *M. edulis *populations of the North Sea and the Bay of Biscay are represented by white squares and white triangles respectively. Sequences sampled in *M. galloprovinciali*s populations of the Atlantic coast of the Iberian Peninsula and Brittany are represented by black diamonds. The tree is rooted by using three sequences from a population of *M. trossulus *of the Baltic Sea (Gdansk, Poland) that are not represented for clarity. Branches in which biggest deletions that explain major changes in the size of *EFbis *alleles are indicated by the reduction in length in base pair (bp). The single sequence of the clade B sampled in an *M. edulis *population is indicated by an arrow.

The reconstructed phylogeny obtained (Figure 4) revealed two highly divergent clades -one was mostly composed of sequences sampled in *M. galloprovincialis *populations (the B clade in Figure 4) and the other was exclusively composed of sequences sampled in *M. edulis *populations (the A clade). The average divergence between sequences of the two clades was 5.2% and the number of fixed differences was 20 (2.2%). The sequence belonging to the B clade sampled in the *M. edulis *population of the Bay of Biscay coalesces with specific alleles at the base of the tree leaving a long interior branch freed from coalescence event (Figure 4). Retention of ancestral polymorphism cannot reasonably explain such a phylogeny. As we paid special attention to refute admixture (see above) we conclude that the presence of this *galloprovincialis *allele in a *M. edulis *sample is a consequence of recent introgression.

**Figure 5:**
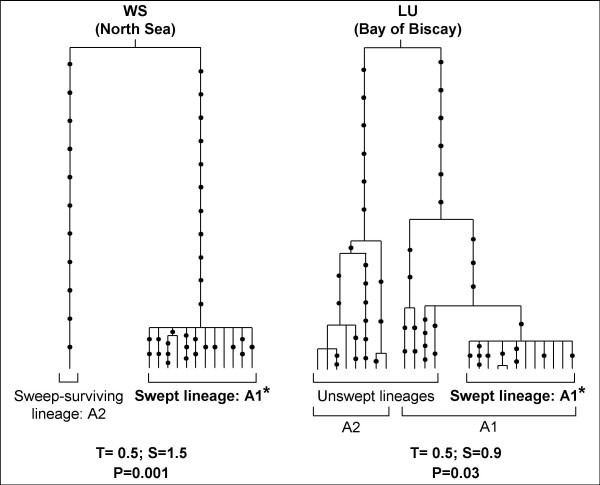
**Parsimoniously reconstructed genealogies of sample WS (*M. edulis*, North Sea) and sample LU (*M. edulis*, Bay of Biscay)**. Mutations are indicated with dots. The single sequence belonging to the B clade sampled in an individual from LU as a result of introgression was used to root genealogies but was not represented and was excluded from the analyses. Interpretations of sample genealogies under the hypothesis of a selective sweep are presented in terms of descendants of the swept allele *versus *ancestral lineages that survived to the sweep through recombination. The results of the likelihood ratio test [16] and estimations of the age, T, and the strength, S, of the sweep are given below genealogies.

In order to facilitate the description of the differences between the two *M. edulis *samples, we split the A clade into two sub-clades, A1 and A2 (Figure 4). Nearly all sequences from the population of the North Sea (WS) belonged to the A1 clade, while half of the sequences from population of the Bay of Biscay (LU) belonged to the A2 clade. This result is more easily visualised in the reconstructed sample genealogies presented in Figure 5.

### Correspondence between length and sequence polymorphisms

**Figure 6:**
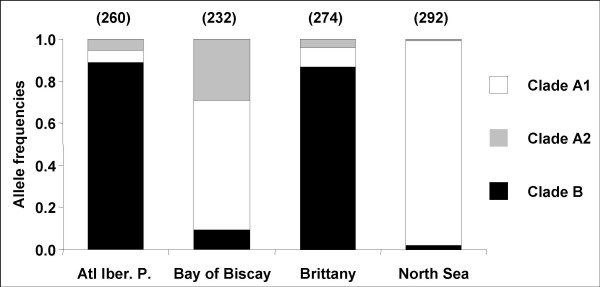
**Allelic frequencies of clades A1 (white), A2 (grey) and B (black) deduced for allele-size frequencies**. Atl. Iberian P.: *M. galloprovincialis *populations of the Atlantic coast of the Iberian Peninsula (samples FA, 1, 2 an 3 in Figure 1); Bay of Biscay: *M. edulis *populations of the Bay of Biscay (samples LU, 4 and 5 in Figure 1); Brittany: *M. galloprovincialis *populations of Brittany (Samples PR, 6, 7 and 8 in Figure 1); North Sea: *M. edulis *populations of the North Sea (samples WS, 9, 10 and 11 in Figure 1). The number of sampled alleles (equivalent to twice the number of individuals) is given within brackets above each histogram.

The results obtained with DNA sequence polymorphisms can be compared to the result obtained with intron-length polymorphism allowing us to infer clade frequencies more reliably with much larger sample sizes. Restricting the sequence analysis to the region amplified with *EFbis *primers, sequences of the B clade were 423 to 427 bp long, sequences of the A1 clade were 446 to 448 bp long and sequences of the A2 clade were 432 bp long. Length polymorphism is the consequence of the existence of many indels, three of which are larger than the others and essentially explain the length difference between clade alleles. Two constantly associated deletions, one of 11 bp and another of 8 bp, explain the small size of alleles of the B clade together with a few other small (< 3 bp) deletions. A deletion of 14 bp explains the intermediate length of alleles of the A2 clade. The precise lengths of *EFbis *size-alleles were previously unknown and we used a relative scale [33]. Sequences now allow us to infer that the three most frequent *EFbis *size-alleles named *G*_0_, *G*_3 _and *E*_0_, have a length of 424 bp, 432 bp and 447 pb respectively, as they pertain to clade B, A2 and A1. We can also deduce that the *EFbis-E*_*i *_alleles are alleles of the A1 clade and that the *EFbis-G*_*i *_alleles are alleles of the B clade with the exception of *EFbis-G*_3 _which corresponds to alleles of the A2 clade. *EFbis-G*_3 _was initially included in the *G*_*i *_size class because it was characteristic of *M. galloprovincialis *progenitors used in a pair-cross experiment between mussels of the Mediterranean and the North Sea [38]. However, Bierne et al. [33] acknowledged such a labelling was not welcomed because population genetics analysis revealed that this allele was indeed characteristic of *M. edulis *populations of the Bay of Biscay. The phylogeny of alleles now removes any doubt and confirms *EFbis-G*_3 _is a distinctive allele of *M. edulis*. According to the correspondence made between the two kinds of data, we present in Figure 6 the inferred frequency of the three clades. Clade B alleles have been sampled at a frequency of 0.2% and 9% in *M. edulis *samples of the North Sea and the Bay of Biscay respectively (Figure 6). Clade A alleles have been sampled at an average frequency of 7% in *M. galloprovincialis *samples (Iberian Peninsula and Brittany). Segregation of heterospecific alleles was therefore detected in every patch although in so low amounts as to be easily overlooked in the DNA sequence samples.

### Departure from neutrality in M. edulis samples

We examined whether the pattern of variation observed at locus *EFbis *within each population of *M. edulis *was consistent with the neutral model at mutation-drift equilibrium. To remove the effect of introgression, the single sequence of the B clade sampled in the population of the Bay of Biscay (LU) was removed from the analysis. The values of Tajima's D [39] and Fay and Wu's H [4] together with their significance are presented in Table 1. A strong departure from mutation-drift equilibrium was observed in the sample of the North Sea (WS). Sequences from WS differ mainly by singleton mutations which results in a star-shaped genealogy (Figure 5) typical of a selective sweep while a single sampled lineage survived to the sweep. Indeed, the significant excess of derived variants at high frequency observed in such a genealogy (i.e. a significant Fay and Wu's test) has been advocated to be a unique pattern produced by hitchhiking [4]. Whilst the same star-like clade (denoted A1* in Figure 5) was also present in sample LU, though in moderate frequency, the departure was not detected by the two tests (Table 1). Indeed, partial sweeps are not easily detected by these two tests [11,40] because they generates an excess of rare and intermediary mutations that compensate in the Tajima's D test and the frequency of derived variants is not high enough to be captured in the Fay and Wu's H test. For instance, Santiago and Caballero [23] showed that positive instead of negative Tajima's D values can sometimes be generated in some subpopulations after a selective sweep in a subdivided population. In order to detect more efficiently the distortion of the distribution in coalescence times in the two sample genealogies we applied the coalescence-based maximum-likelihood method of Galtier et al. [16]. We were able to easily apply this method to our data as they were compatible with the infinite mutation model (no recombination, Table 1). For the two populations, the model with a recent reduction in effective size was significantly better supported than the mutation-drift equilibrium model (Likelihood ratio tests: p = 0.001 and p = 0.03 for samples WS and LU respectively). However, the estimated strength of this effect was stronger in the North Sea (S = 1.5 coalescence time units) than in the Bay of Biscay (S = 0.9 coalescence time units) while the estimated age of the event was roughly the same in the two samples (T~0.5 coalescence time units). These results provide statistical support to what can be easily visualised in the reconstructed sample genealogies presented in Figure 5.

### Genetic hitchhiking past a barrier to gene flow

We hypothesised that the past fixation of the same favourable mutation could have been responsible for the distortion of allele genealogies in the two *M. edulis *patches, a hypothesis on which we will come back in the Discussion section. Our analyses suggest that the distortion of allele genealogies and allele frequencies is less pronounced in the patch of the Bay of Biscay than in the patch of the North Sea. Although few models of genetic hitchhiking in subdivided populations are available, they have emphasised that this situation could produce spatial variation in genetic diversity even without spatial variation in selection regimes [23]. In order to illustrate this effect in a context that matches our study system we modelled the hitchhiking effect of a favourable mutation that crosses a barrier to gene flow (see methods). We considered two patches of hundred of demes. Within patches, a stepping stone of demes were connected by an appreciable level of migration (*m *> 0.1), while the migration rate between patches was much smaller (*m*_*bar *_<< 0.1). Our model therefore resembles the model of Barton [22] within patches and the model of Slatkin and Wiehe [21] between patches.

**Figure 7:**
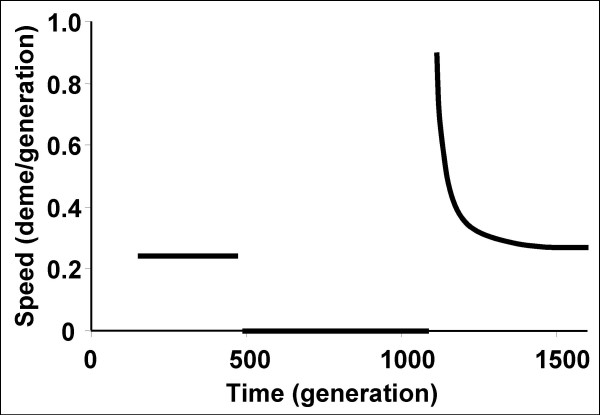
**Speed of the centre of the wave of advance of a favourable allele when crossing a barrier to gene flow**. Results from a linear one-dimensional stepping-stone model with *p*_0 _= 0.0001, *m *= 0.3, *s *= 0.1 and *m*_*bar *_= 10^-8^. Two hundred and fifty demes were used with a barrier to the right of deme 100.

The behaviour of the favourable mutation itself has already been described by Pialek and Barton [41]. We obtained the same results with our deterministic simulations. Indeed, positive selection generates a Fisherian wave of advance of the selected allele that spreads through the metapopulation at a constant speed (proportional to migration and selection) until it reaches the barrier to gene flow. After a delay which depends on the strength of the barrier, the favourable allele inevitably crosses the barrier. The relation between the delay and the strength of the barrier is logarithmic and very strong barriers are required to produce appreciable delays [41]. Once the favourable allele finally crosses the barrier the wave of advance forms again but transiently accelerates (Figure 7). This surprising behaviour has been described and discussed by Pialek and Barton [41]. The wave is broadened by the barrier and hence transiently spreads faster than a Fisherian wave and later progressively slows down to its equilibrium speed while recovering a Fisherian shape [41].

**Figure 8:**
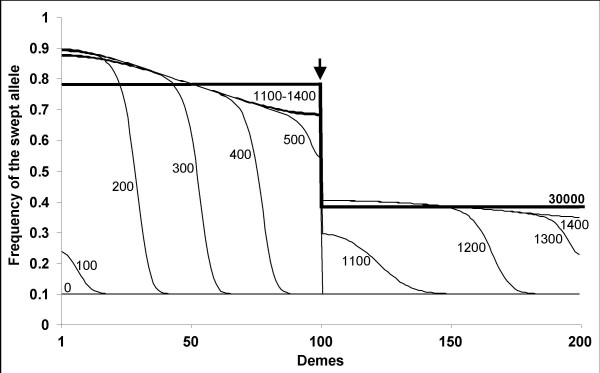
**Genetic hitchhiking past a barrier to gene flow**. Frequency of the neutral allele A initially linked to the favorable allele. Generations during which allele frequencies were recorded are indicated next to the curves. Results from a linear one-dimensional stepping-stone model with *p*_0 _= 0.0001, *m *= 0.3, *s *= 0.1, *c *= 0.01 and *m*_*bar *_= 10^-8^. Two hundred demes were used with a barrier to the right of deme 100. The position of the barrier is indicated by a vertical arrow. Note the steplike discontinuity in clines at the barrier where all curves are vertical.

We now investigate the effects of the sweep at the selected locus on neutral variation at a linked locus. We first describe the process with a set of parameters that produces the effect sought. In the left patch in which the favourable B allele originates within the first left deme, the neutral A allele initially linked with B takes the lift and follows the wave of advance (Figure 8). However, recombination breaks the hitchhiking effect – the A allele does not reach fixation in any deme and its rise in frequency is gradually attenuated. The selective sweep thus generates a gradient in allele frequency (Figure 8). After the delay required for the favourable allele to cross the barrier has elapsed, hitchhiking starts again on the other side of the barrier, in the right patch. However, the effect is lessened in proportion to the strength of the barrier. The rise in allele frequency being slighter, a step in allele frequency is generated (Figure 8). In other terms, the delay imposed by the barrier to the spread of the favourable allele represents an additional time window for recombination to act and dissociation between the neutral allele and the selected allele to occur. Once the sweep is finished, migration homogenises allele frequencies within each patch leading to flat curves while the barrier slows down homogenisation between patches. The consequence is to further increase the step in allele frequency between the two patches.

**Figure 9:**
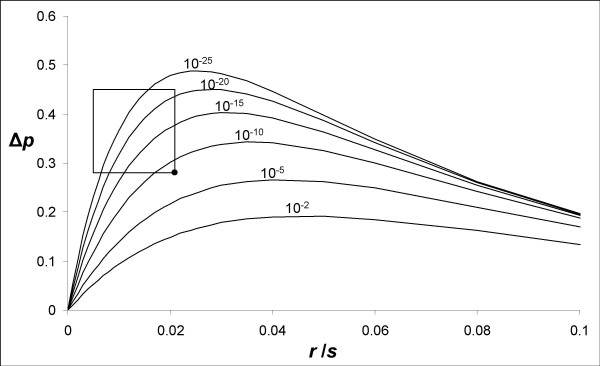
**Magnitude of the step in neutral allele frequency between the two patches (Δ*p*) plotted against *r*/*s *for different strength of the barrier**. Barrier strengths (*m*_*bar*_) are indicated above each curve. The rectangle indicates the parameter space that could explain the results observed at the *EF1α *gene in *M. edulis*, and the dot shows the corner of that space which corresponds to the smallest barrier strength required. Results from a linear one-dimensional stepping-stone model with *p*_0 _= 0.0001, *m *= 0.3. Two hundred demes were used with a barrier to the right of deme 100.

Under our deterministic model, the magnitude of the effect mainly depends on two parameters: the *r*/*s *ratio (as in any hitchhiking model [22]) and the strength of the barrier [29]. The number of demes and the migration rate between demes within patches has an effect on the gradient in allele frequency within patches and therefore mainly affect the second phase once the sweep is finished but the step in allele frequency still increases because allele frequencies homogenise in each patch. However, the effect of these two parameters is secondary to the barrier strength and *r*/*s*. In Figure 9 is presented the magnitude of the step in neutral allele frequency between the two patches, Δ*p*, produced at the end of our simulations (when homogeneity is reached within patches) as a function of *r*/*s *for various barrier strengths. When *r*/*s *is very small, the A allele reaches high frequencies everywhere, even behind the barrier in the second patch, and Δ*p *is small. When *r*/*s *is large, the hitchhiking effect is weak everywhere, even before the barrier in the first patch, and Δ*p *is also small. For intermediate values of *r*/*s*, appreciable Δ*p *values can be produced providing the strength of the barrier is strong enough. The relation with the strength of the barrier appears to be logarithmic, as is the delay of crossing [41]. To summarise, our simple model predicts that substantial population structure can be generated when a strong barrier exists and the *r*/*s *ratio takes intermediate values.

## Discussion

Our eleven-locus F_ST _scan of the *Mytilus edulis *genome provided clear results: one locus, *EFbis*, behaved very differently from the other 10 loci (Figure 2A) and the outlier test of Beaumont and Nichols [25] allowed us to validate the impact of selection on this locus (Figure 2B). The approach of scanning genomes for F_ST _outliers is becoming a standard in molecular ecology [42] and the number of studies that inferred candidate loci for adaptation through this approach is increasing rapidly [43-45]. These studies often used molecular markers that do not easily allow reconstructing the genealogical relationship among alleles, such as microsatellites or AFLPs. Although selected loci are discovered, one can get little information on the exact form of selection that is/was responsible for the discrepancies observed at these loci. DNA sequence polymorphism allows a more precise analysis of selective effects but the approach of scanning genomes for regions of low nucleotide diversity [17,31] is rarely simultaneously conducted with F_ST _scans. Few studies have pursued the analysis of an F_ST_-outlier locus by a more refined analysis of DNA sequence variation [30,46]. The results we here obtained in the analysis of DNA sequence polymorphism at an F_ST_-outlier locus were in remarkable agreement with the F_ST_-scan approach as we observed a clear departure from mutation-drift equilibrium in the form of a clade of alleles with a star-shaped genealogy (clade A1* in Figure 5). We are now in a position to discuss the various possible selective scenarios which could have created the pattern of differentiation observed.

### The genetic structure observed at the EF1α gene between M. edulis populations is not a consequence of differential introgression

When we undertook the analysis of DNA sequences at the *EF1α *gene we had in mind the hypothesis of adaptive introgression of a *galloprovincialis *allele within the *M. edulis *patch of the Bay of Biscay. We are now in position to dismiss this interpretation, as our data establish that introgression does not interfere with differentiation between *M. edulis *populations at the *EFbis *locus. The phylogenetic analysis of DNA sequence polymorphism revealed the strong divergence that exists between alleles of the two species and only a single introgressed allele has been sampled (Figure 4). The analysis of allele frequencies allowed us to study more precisely introgression levels. Although introgression appeared to be slightly stronger in the enclosed patch of the Bay of Biscay than in the Northern peripheral patch, introgression is low in both patches and does not perturb the analysis of the differentiation between the two *M. edulis *patches.

### Indirect hitchhiking selection

In this section, we would like to settle whether selection acted directly on the locus surveyed or indirectly on a linked locus. The *EF1α *gene is recognised to be under strong purifying selective constraints and is not a good candidate for short term adaptive evolution. In addition, the few polymorphic non-synonymous mutations observed were in low frequency (singletons or doubletons). Furthermore, as we did not detect recombination in our data, the hitchhiking effect of a favourable mutation localised within the sequence surveyed should have eliminated all linked variation and produced a perfect star genealogy on which new post-hitchhiking mutations would map. In the presence of recombination between a selected locus and the locus studied, hitchhiking should have been incomplete and ancestral lineages could have survived to the sweep producing a partially star-shaped genealogy [4]. The genealogies obtained, in which such sweep-surviving lineages have been sampled (Figure 5) are therefore in much better agreement with the hypothesis of indirect selection.

As selection must have been indirect, an inevitable question is the chromosomal distance that separates the selected locus and the locus surveyed. With the data at hand we cannot answer precisely this question but can try to give some indications. The chromosomal length affected by a selective sweep mainly depends on the strength of selection, the recombination rate, the population size, and the time elapsed since the sweep [47-49]. Here, the sweep would be young enough for a quasi absence of younger coalescence than the multifurcated one produced by the sweep (Figure 5). The population size, *N*, is negatively correlated with the magnitude of the hitchhiking effect. Given the very large population size for *M. edulis*, one may argue that a selective sweep would not have effects extending very far on either side of the adaptive substitution which may therefore possibly belong to unsequenced portion of the *EF1α *gene itself. However, thorough investigations of the effect of *N *on the hitchhiking effect showed that it is not as large as that determined by the *r*/*s *ratio [6,49]. In fact, the main effect of *N *is to determine *p*_0 _the initial frequency of the favourable mutant (*p*_0 _= 1/2*N*), and was already incorporated in the deterministic model of Maynard Smith and Haigh [3], while the effect of a finite population size is negligible as soon as *N *is not too small [49]. In the single population deterministic model, the final frequency after the sweep, u*, of the neutral allele A that hitchhikes with the favourable mutation is:

(1) u* = *u*_0 _+ (1-*u*_0_) *p*_0 _^*r*/*s*^

where *u*_0 _is the initial frequency of A and *p*_0 _the initial frequency of the favourable mutation [22]. Taking into account the gene diversity observed in the *M. galloprovincialis *sample (Hd = 0.99) that has not been influence by hitchhiking selection, one can easily assume *u*_0 _to be small and simply approximate equation 1 by:

(2) u*~(2*N*)^-*r*/*s*^

Equation 2 allows us to have an idea of the *r*/*s *ratio required to produce the frequency of A1* allele observed in the sample of the North Sea (WS), which was ~0.9. For instance, *r*/*s *would need to be 0.015 if *N *= 10^3^, 0.008 if *N *= 10^6^, 0.006 if *N *= 10^8^. Although we have no idea of the local recombination rate in the region of the mussel genome in which *EF1α *is located, we know that the recombinational size of the *M. edulis *genome is ~1000 cM [50] and the physical size is ~1500 Mb [51], which leads an average recombination rate of 0.7 cM/Mb (as an indication, the same calculation in *Drosophila melanogaster *would give 1.5 cM/Mb). The distance, *d*, in Kb that separates the *EFbis *locus and the selected locus is therefore estimated to be ~1000*s *(e.g. 1 Kb when *s *= 0.001, 100 Kb when *s *= 0.1).

### Selective scenarios: contemporary local adaptation versus past unconditional positive selection

Usually high F_ST _outliers are interpreted as being the consequence of local adaptation [25,42,52]. The simplest form is disruptive selection on a bi-allelic locus in a two-habitat model, one allele being favoured in one habitat and the other allele being favoured in the other habitat. It has been shown that local selection produces higher F_ST _values than expected without, and footprints on the diversity at linked neutral loci [20,43]. Under such a scenario, the derived favoured mutation at the selected locus responsible for the star-shaped clade of alleles at the neutral locus (A1* in Figure 5) would have been fixed by positive selection in populations of the North Sea only while being counter-selected in populations of the Bay of Biscay. The presence of alleles of the clade A1* in the enclosed patch of the Bay of Biscay would be a consequence of recombination and gene flow. However, our data revealed many A1* alleles in the Bay of Biscay and very few A2 alleles in the North Sea. In the local adaptation scenario, this would imply asymmetric effects of recombination and neutral gene flow (predominantly from the North Sea to the Bay of Biscay but not the reverse), which are hard to explain. Besides, although some may inevitably point to some environmental differences between the North Sea and the Bay of Biscay (e.g. temperature), it is not clear that these differences should be more pronounced that the environmental heterogeneity observed within each patch.

We therefore hypothesised that the same favourable mutation had gone to fixation in the two populations [29], although this hypothesis is rarely considered to explain high F_ST_s. The interest of this hypothesis is to be inherently asymmetric because the wave of advance of a favourable allele is directional from its patch of origin to the other. The simple model we developed to illustrate this scenario together with the few available models of genetic hitchhiking in subdivided populations [21-23] show that the hitchhiking effect is expected to diminish as the favourable mutation spreads from the deme in which it originates. The presence of a barrier to gene flow amplifies the effect (Figure 8) owing to the delay produced to the spread of the favourable allele and to the peculiar behaviour of a wave of advance when crossing a barrier [41]. This would explain the moderate frequency of clade A1* and the persistence of unswept alleles (e.g. clade A2) in the Bay of Biscay. The genealogy presented in Figure 5 fits very well with a genealogical interpretation of the model. Although our model is bi-allellic, one simply has to imagine that the unswept allele, a, is composed of old lineages that survived the sweep (non-A1* sequences) and the swept allele, A, is composed of young lineages belonging to the star genealogy (A1* sequences). The moderate frequency of the swept allele would also explain why statistics that summarise the mutation frequency spectrum such as Tajima's D and Fay and Wu's H did not capture the departure from the standard coalescent in the second patch [4], although methods that incorporate the additional information of linkage disequilibrium can [16]. Furthermore, the delay required for the wave to cross the barrier should result in a younger coalescence (smaller terminal branches) of the lineages affected by the selective sweep (A1*) in the patch of the Bay of Biscay, a tendency that is actually observed in Figure 5. However, the barrier needs to be strong in order to generate the observed step in allele frequency, Δ*p*~0.4 (Figure 9, also see [21]). Furthermore, we have seen that the *r*/*s *ratio needed to be small in order to generate a high frequency of A1* allele in the North Sea (0.005 <*r*/*s *< 0.02). Combining the two observations allows deducing that the migration rate between the two patches needs to be lower than *m*_bar_~10^-8 ^(dot in Figure 9).

A necessary hypothesis of the hitchhiking in space scenario is that selection should be a stronger force than genetic drift. Otherwise the barrier to gene flow between the two patches of *M. edulis *would produce detectable F_ST _at other neutral loci than *EFbis*. This assumption is present in the models of Slatkin and Wiehe [21] and ours that are deterministic. To employ the terminology developed by Gillespie [14], genetic drift should be a minor force relative to genetic draft, the impact of indirect selection on neutral variability. The scenario proposed would therefore mainly concern species with large effective population sizes. Although their effective size has often been debated [53], marine bivalves are known to reveal among the highest levels of genetic diversity ever observed within the animal kingdom [54-56]. Levels of diversity observed in *Mytilus *are in accordance with this view (Table 1). Indeed, marine bivalves are good candidates for the genetic draft model as was established for mitochondrial DNA [55] and suspected for nuclear genes [57].

Another question that emerges is the nature of the barrier to gene flow. A first possibility is geographic isolation, as is observed nowadays. The enclosed situation of the *M. edulis *patch of the Bay of Biscay isolates it from peripheral populations. Assuming that the local biogeography (*i.e*. the mosaic structure observed nowadays) has been stable for a while, the two *M. edulis *patches might be connected only by very rare events of long range dispersal. Alternatively, one can imagine that the favourable mutation has had to transit through the *M. galloprovincialis *genomic background before reaching the enclosed patch of *M. edulis*. Under this scenario the mutation would need to be neutral or slightly deleterious in the *M. galloprovincialis *background although favourable in the *M. edulis *background. It is also possible that the sweep predates the mosaic structure observed nowadays. We would therefore need to consider the possibility that the barrier was genetic instead of physical. The genetic barrier would simply have come to coincide secondarily with a region of low dispersal as theoretically expected [58]. In a sense considering a genetic barrier amounts to consider the first scenario again -that the two *M. edulis *populations are structured into two backgrounds in such a way that at least in the genomic region of the *EF1α *gene, mixtures of genes from North Sea and Bay of Biscay are counter-selected. However, even if divergent selection occurs in the background, a neutral allele of the screened locus is assumed to have hitchhiked with an unconditionally favourable mutation from its background of origin to the other.

## Conclusion

To conclude, we have shown that, in a structured population, a selective sweep at a positively selected linked locus is a simple scenario to account for unusually high level of differentiation at a marker locus. This scenario has rarely been considered in the literature [29] and likely applies to the example of the *EF1α *gene in *M. edulis*. However we cannot completely exclude more complex scenarios of local adaptation, whereby the selected allele responsible for the selective sweep would be confined in its patch of origin. A decisive (though very demanding) test would require walking on the chromosome toward the direct target of selection: under the scenario of local adaptation F_ST _should increase [43] while under the scenario of unconditional positive selection F_ST _should decrease (Figure 9, [29]).

Distinguishing the two scenarios ('local adaptation' and 'hitchhiking in space' [29]) is not unnecessary subtlety, it has important consequences on our appreciation of the cost paid by species to adapt to local environmental variation. It is very different to conclude that ~10% (one of eleven randomly chosen loci) of the genome has been marked by the footprint of past positive selection or ~10% of the genome is affected by a polymorphism maintained by selection in a heterogeneous environment. This is the reason why the 'hitchhiking in space' hypothesis should be considered more closely in genome scan studies.

## Methods

### Sampling

On the basis of previous publications, we selected four geographic samples of 48 individuals to represent each of four species patches: FA (Faro, Algarve, Portugal) and PR (Primel, Brittany, France) respectively representative of *M. galloprovincialis *populations of the Atlantic Coasts of the Iberian Peninsula and Brittany, and LU (Lupin, Charente-Poitou, France) and WS (Wadden Sea, Holland) respectively representative of *M. edulis *populations of the Bay of Biscay and the North Sea (Figure 1). The PR sample was described in Bierne et al. [33]. Samples FA and LU are new samples collected in the same sites as samples Faro and Brouage described in Bierne et al. [33]. Sample WS is a new sample collected in the middle of a well characterised peripheral patch of the hybrid zone, well away from the transition zones. Additionally, we used a sample of *M. trossulus *(GD) from Gdansk (Poland) in the Baltic Sea as an outgroup. The new samples were treated as previously described [33,59] except that we used the phenol-chloroform protocol to extract genomic DNA rather than the Chelex protocol.

### Outlier tests

In order to test that the level of differentiation observed at the *EFbis *locus was significantly higher than the level of differentiation observed at other loci between the peripheral *M. edulis *population of the North Sea and the internal patch of the Bay of Biscay, we compiled data on 5 allozyme loci (*EST D, LAP, PGI, OCT, MPI*) from Coustau et al. [34] and 6 nuclear DNA loci (*EFbis, mac-1, Glu5', DAMP1, DAMP2, DAMP3*) from Bierne et al. [60]. The patch of the Bay of Biscay was represented by samples "Noirmoutier" and "Royan" of Coustau et al. [34] for allozyme loci and samples "Brouage" and "Boyar" of Bierne et al. [60] for nuclear DNA markers. The patch of the North Sea was represented by samples "Caen" and "Danemark" of Coustau et al. [34] for allozyme loci and samples "Grand-Fort-Philippe", "Tichwell" and "Cley" of Bierne et al. [60] for nuclear DNA markers. We first depicted the diversity observed in each patch at each locus by computing unbiased gene diversities [61] and estimating their confidence intervals by permutation techniques with the Genetix 4.0 software [62]. Secondly, we used the method of Beaumont and Nichols [25] to identify loci that depart from the expected neutral distribution of F_ST_. The average and 95% confidence interval of single locus F_ST _as a function of heterozygosity was obtained from simulations performed with the fdist2 program. In order to be as conservative as possible, we used several set of parameters (number of demes, mutation model etc.) and used parameters that maximized the upper limit of the 95% confidence interval.

### Analysis of the genetic composition of samples

The four newly sampled populations were analysed at the same three loci used by Bierne et al. [33], *EFbis*, *mac-1 *and *Glu-5'*. Polymerase chain reaction (PCR) and electrophoresis were performed as previously described. The fluorescent dye 5' end-labelled-primer technique was used, with dye 6-FAM (Sigma Genosys) for the forward primer of *mac-1 *and the primer *Me15 *of *Glu-5' *and TAMRA (Sigma Genosys) for the primer *EFbis-F*. Gels were scanned in a FMBIO II fluorescence imaging system (Hitachi Instruments) at 505 and 605 nm.

We performed a correspondence analysis (CA) on the matrix of allele counts per sample using the Genetix 4.0 software [62]. This CA was performed in order to verify that the genetic composition of the four samples chosen for DNA sequencing matched the expectation from their geographic location. We used 11 previously analysed samples chosen to be representative of the four geographic patches: Faro (a previous sample from Portugal performed at the same location as sample FA), Setubal, Biarritz, Brouage, Boyard, Moguériec, Morlaix, Polzeath, Grand-Fort-Philippe, Cley, Tichwell [33]. In addition, homogeneity of allelic frequencies between pairs of populations was tested by an exact test using the Genepop software [63] which allowed us to group samples with the same genetic composition. Finally, departure from Hardy-Weinberg and linkage equilibrium was tested with the Maximum-likelihood method of Barton [35] using species-specific compound alleles as described in Bierne et al. [33].

### DNA polymorphism

DNA sequences were obtained on a longer fragment of the *EF1α *gene than the *EFbis *locus, including the full region screened for length polymorphism. The new locus named *EFseq *was approximately one kilobase long (the shortest observed allele was 1013 bp long and the longest observed allele was 1349 bp long) and includes the second and the third introns, the third exon and portions of the second and fourth exons of the *EF1 α *gene. *EFseq *was amplified with the same reverse primer as *EFbis*, *EFbis*-*R *[38], which was designed in the fourth exon of *EF1α*, and a newly designed forward primer, *EFseq-F *(5'-AGGCTCCTTCAAGTACGCCTGGG-3'), designed in the second exon. The protocol of PCR reactions was the same as the one described for the *EFbis *locus [38] except that the annealing temperature was increased to 60°C and the elongation step was increased to one minute. The following steps -which include purification of PCR products, cloning and sequencing- were done following the mark-recapture (MR)-cloning protocol described in Bierne et al. [36]. Briefly, MR-cloning allowed us to perform a single cloning reaction per population samples. Each individual of a sample were PCR-amplified separately using 5'-tailed primers with small poly-nucleotide tags. PCR products of similar quantities were mixed together and cloned into a pGEM-T vector by using a Promega pGEM-T cloning kit (Promega, Madison, WI, USA). Clones were sequenced with universal plasmid primers. The individual from which a sequence comes was identified by the tag sequences upstream of each initial primer. A consequence of the MR-cloning protocol is that the sample size is not completely under control. Within a given number of sequenced clones, the same allele of the same individual (recognised using the nucleotide tag) can be cloned several times, while some alleles or individuals are absent. Therefore the number of different sequences obtained is less than, although positively correlated to, the number of positive clones sequenced (called the effort of capture in [36]). However, an interesting side-effect of this protocol is the opportunity to assess the error rate due to mutations during the cloning and amplification process. Singleton mutations (which are important indicators of selective or demographic effects) are particularly sensitive to such artefacts; by restricting the analysis to sequences that were captured twice or more for the same individual, one can assess the potential impacts of artefacts [36].

### Sequence analyses

Sequence alignment was performed with ClustalW [64] in the BioEdit interface [65] and verified by eye. For each sequence, the size of the region corresponding to the *EFbis *locus was computed. Alignment gaps were then excluded from the analyses. To give a representation of allele genealogy, phylogenetic reconstructions were obtained with Mega 3.1 [66] using the neighbour-joining (NJ) algorithm with number of nucleotide differences. We used DNAsp [67] to compute basic population genetic parameters: the number of polymorphic sites, the number of synonymous, non-synonymous and non-coding mutations, levels of nucleotide diversity estimated from the number of polymorphic sites, θ_W _[68], or from pairwise differences, θ_π _[37], and the minimum number of recombination, R_M_, estimated by the method of Hudson and Kaplan [69]. DNAsp was also used to compute some indicators of the distortion of the allele frequency spectrum from the neutral expectation at mutation-drift equilibrium. Tajima's D [39] is a well-known statistic that proved very efficient to detect a shift of the allele frequency spectrum towards low-frequency polymorphism. The Fay and Wu's H test [4] is a more recent improvement that focuses on high frequency derived mutations, an excess of which is a specific footprint of a selective sweep. Departure from the neutral expectation at mutation-drift equilibrium was tested by coalescent simulations without recombination conditional on the number of segregating sites [70] that here proved to be the most conservative procedure. To the summary statistic approaches we added the coalescence-based maximum-likelihood method of Galtier et al. [16]. This method is designed to detect a distortion in the shape of gene genealogies generated by a diversity-reducing event (hitchhiking or bottleneck). The likelihood of a model in which a drop in effective size of strength S occurred at time T in the past is compared to the likelihood of a constant-size model. Galtier et al. [16] defined S as the time that would be required to generate the same amount of coalescence if the population size had not changed. S is therefore expressed in coalescent time units (*i.e*. units of 2*N*_*e *_generations).

### Deterministic model of genetic hitchhiking in a subdivided population

We developed a simple simulation model in order to illustrate the impact of a barrier to gene flow on the hitchhiking process. We modelled the fixation of an advantageous mutation and its effect on a linked neutral locus in large populations with a deterministic approach [3,21]. We used a classical linear one-dimensional stepping-stone model composed of 200 demes. The migration rate between demes was *m *(*m*/2 in either direction). A barrier to gene flow was positioned in the middle of the metapopulation. The migration rate was *m*_*bar *_(*m*_*bar *_<<*m*) between deme 100 and deme 101. We considered two bi-allelic haploid loci with recombination rate *c *between them. One locus was neutral and the other was under positive selection. At the selected locus, allele B has a selective advantage *s *over the alternative allele b. Initially, all the demes were fixed for b and allele B was introduced in the first deme on the left side of the chain at a frequency *p*_0_. At the neutral locus, we assumed that one allele, A, was initially in frequency *u*_0 _in all the demes of the chain (the other allele, a, being at frequency 1-*u*_0_). Allele B at the selected locus was initially on a chromosome carrying A at the neutral locus. Genotypic frequencies in each deme at a given generation are deduced from the frequencies of the previous generation after accounting for migration, recombination and selection. We registered the evolution of allele frequencies and the speed of the wave of advance. To calculate the speed, the centre of the wave was defined as the deme in which the B allele frequency was closer to 0.5. A Borland Delphi 4.0 program is available from the authors upon request.

## Authors' contributions

MFF carried out the molecular genetic studies, analyzed the data and drafted the manuscript as part of his PhD dissertation. PD and FB provided advises for data analysis and interpretation, and helped to draft the manuscript. NB conceived, initiated and coordinated the study, analyzed the data, wrote the programme for deterministic simulations and drafted the manuscript. All authors have read and approved of the final manuscript.
